# The Role of Non-Coding RNA in the Pathogenesis of Hypertensive Nephropathy

**DOI:** 10.3390/cells15080701

**Published:** 2026-04-15

**Authors:** Paulina Plewa, Karolina Figiel, Maciej Ćmil, Patryk Skórka, Kacper Kupis, Andrzej Pawlik

**Affiliations:** Department of Physiology, Pomeranian Medical University, 70-111 Szczecin, Poland; paulina.plewa@op.pl (P.P.); kfigiel344@gmail.com (K.F.); cmilmaciej@gmail.com (M.Ć.); p.skorka04@gmail.com (P.S.); 93210@student.pum.edu.pl (K.K.)

**Keywords:** hypertensive nephropathy, non-coding RNA, microRNA, long non-coding RNA, circular RNA

## Abstract

Hypertensive nephropathy (HN) is a leading cause of chronic kidney disease and end-stage renal disease worldwide and results from the long-term effects of hypertension on renal structure and function. The pathogenesis of HN is complex and involves haemodynamic disturbances, renal vascular injury, oxidative stress, chronic inflammation, and progressive interstitial fibrosis. In recent years, increasing attention has focused on the role of non-coding RNAs (ncRNAs)—including microRNAs (miRNAs), long non-coding RNAs (lncRNAs), and circular RNAs (circRNAs)—as key regulators of gene expression involved in these processes. This review summarises the current understanding of the molecular mechanisms underlying HN, with particular emphasis on the roles of oxidative stress, activation of the renin–angiotensin–aldosterone system, transforming growth factor beta signalling, and inflammatory and fibrogenic pathways. The contribution of dysregulated ncRNAs to endothelial dysfunction, inflammatory responses, apoptosis, angiogenesis, and renal remodelling and fibrosis is also discussed. Particular attention is given to miRNAs and lncRNAs as mediators of disease progression and potential biomarkers, as well as to the emerging role of circRNAs in hypertensive kidney injury, including their involvement in the regulation of redox balance and intercellular communication. Collectively, available evidence indicates that ncRNAs represent a critical link between haemodynamic stimuli and persistent molecular alterations in renal tissue, highlighting their potential as diagnostic markers and therapeutic targets in HN.

## 1. Introduction

Hypertensive nephropathy (HN) is a chronic kidney disease (CKD) that develops as a consequence of long-term arterial hypertension (AH) and is characterised by progressive structural and functional renal damage. Persistently elevated blood pressure leads to renal vascular remodelling, glomerulosclerosis, and interstitial fibrosis, which may result in the development of CKD and end-stage renal disease (ESRD). HN represents one of the leading causes of renal failure worldwide and significantly contributes to increased cardiovascular morbidity and mortality [1]. The epidemiological burden of HN is substantial and continues to increase due to population ageing, the rising prevalence of hypertension, and the coexistence of metabolic disorders. Hypertension-related kidney damage is a major contributor to CKD globally and accounts for a significant proportion of patients requiring renal replacement therapy. This condition generates considerable healthcare costs and negatively affects patients’ quality of life, leading to premature mortality. In recent years, non-coding RNAs (ncRNAs) have been recognised as key regulators of gene expression and cellular homeostasis, playing important roles in numerous biological and pathological processes. The main classes of ncRNAs include microRNAs (miRNAs), long non-coding RNAs (lncRNAs), and circular RNAs (circRNAs), which regulate gene expression through transcriptional, post-transcriptional, and epigenetic mechanisms. ncRNAs regulate multiple molecular pathways, including cell proliferation, inflammatory responses, apoptosis, and fibrotic processes, which are crucial in the pathogenesis of kidney diseases. Increasing evidence indicates that dysregulated ncRNA expression contributes to the initiation and progression of HN by modulating vascular function, oxidative stress, immune responses, and fibrogenic signalling pathways. Because of their stability in body fluids and tissue specificity, ncRNAs are considered promising biomarkers for the early diagnosis and prognosis of kidney diseases. Furthermore, a growing body of research suggests that ncRNAs play a central role in regulating complex gene networks associated with renal injury, making them key molecular components in the pathogenesis of HN [2]. This review summarises recent and influential publications on the role of ncRNAs in the pathogenesis of HN and the molecular mechanisms by which they regulate the processes leading to kidney damage (e.g., [1] or [2,3], or [4,5,6]; see the end of the document for further details on references).

## 2. Pathophysiology of HN

### 2.1. Renal Vascular Injury in Hypertension

Hypertension causes chronic injury to renal vessels and is a core driver of nephrosclerosis and the progressive decline in renal function [3,4]. Lesions in afferent arterioles and small arteries (arteriolosclerosis/arteriosclerosis) predominate, increasing vascular resistance, impairing glomerular protection against systemic pressure fluctuations, and promoting downstream ischaemia [3]. Accordingly, vascular damage in hypertension is not only a background contributor to glomerular lesions but can directly disturb single-nephron haemodynamics and induce microvascular hypoxia within the interstitium [3]. Hypertension also accelerates arteriosclerotic remodelling, including intimal thickening driven by smooth muscle cell phenotypic switching, migration into the intima, and increased collagen deposition that collectively thicken the vessel wall [4]. These structural changes contribute to nephrosclerosis and may reduce renal flow reserve, increasing vulnerability to ischaemic injury during pressure fluctuations or heightened oxygen demand. Clinically, limited flow reserve may partly explain why some patients experience a decline in kidney function during intensive blood pressure lowering, particularly when baseline renovascular disease is advanced [3]. Early in remodelling, cellular reorientation and wall hypertrophy can increase stiffness with relatively modest luminal narrowing, potentially delaying recognition of progressive microvascular dysfunction. Consistent with this concept, chronic exposure of preglomerular vessels to elevated pressure produces a slowly progressive pathology typical of ‘benign’ nephrosclerosis [5]. A common coexisting lesion is arteriolar hyalinosis, characterised by a loss of smooth muscle cells and deposition of hyaline material within the vessel wall [4]. Histologically, hyalinosis appears as eosinophilic, amorphous material in the arteriolar wall and is distinct from arteriolosclerosis sensu stricto [4,6]. Seccia et al. reported thinning of the media with focal accumulation of hyaline material in areas of smooth muscle loss in hypertension. Functionally, the loss of contractile elements impairs vasomotor control and autoregulation, while increased endothelial permeability facilitates plasma protein leakage into the vessel wall, perpetuating injury [4]. Hyalinosis can be observed with ageing; however, in hypertension, it tends to occur earlier and be more severe [5]. Beyond changes in the media and intima, hypertensive vascular injury includes endothelial dysfunction that alters vascular tone, permeability, inflammation, and the local prothrombotic milieu [7]. Within the renal microcirculation, peritubular capillary rarefaction is particularly important because it limits oxygen delivery to tubules and aggravates hypoxia [8]. Li et al. emphasised that microvascular loss accelerates CKD progression through endothelial dysfunction, hypoxia, inflammatory and oxidative signalling, and subsequent fibrosis [9]. Damage to the endothelial glycocalyx may represent a mechanistic link between endothelial dysfunction and impaired autoregulation, as the glycocalyx functions as a mechanosensor coupling shear stress to endothelial nitric oxide synthase (eNOS) activation and nitric oxide (NO) production [6]. Ito and Mori proposed that glycocalyx degradation could weaken the myogenic response of the afferent arteriole, promoting glomerular barotrauma as well as an ischaemic phenotype once luminal stenosis becomes significant [6]. Another review highlighted that glycocalyx injury exposes adhesion molecules (e.g., intercellular adhesion molecule and vascular cell adhesion molecule), facilitates leukocyte adhesion and platelet aggregation, and fosters an inflammatory–thrombotic response in the microcirculation [8]. In extreme hypertensive phenotypes, particularly malignant hypertension, rapid small vessel injury with hyperplastic (‘onion skin’) arteriolosclerosis and fibrinoid necrosis is typical. Haridasan et al. summarised the classic renal histopathology of malignant hypertension, including endothelial oedema, thrombotic microangiopathy, and fibrinoid necrosis with mucoid intimal hyperplasia. Notably, in malignant hypertension-associated thrombotic microangiopathy, lesions may be more prominent in arteries and arterioles than within glomerular tufts themselves [10]. A systematic review by Maisons et al. reported that severe arteriolar injury is very common in malignant hypertension and that renal thrombotic microangiopathy is present in the majority of biopsies. That review also underscored frequent diagnostic heterogeneity in biopsy cohorts, implying that malignant hypertension may overlap with other kidney diseases and thereby compound vascular injury [11].

### 2.2. Oxidative Stress

Oxidative stress is a key factor in hypertension and the renal damage that accompanies it; importantly, this relationship is bidirectional, with oxidative stress acting as both a driver and a consequence of disease processes [12,13,14]. During hypertension, excessive production of reactive oxygen species (ROS), including superoxide (O_2_^−^) and hydrogen peroxide (H_2_O_2_), occurs largely due to activation of nicotinamide adenine dinucleotide phosphate (-) oxidases (NOX1, NOX2, NOX4) in the kidney and vasculature [12,13,14,15,16]. ROS can scavenge NO, promoting endothelial dysfunction and increased renal vascular tone, thereby contributing to higher peripheral resistance and sustained hypertension [14]. Elevated ROS also activates redox-sensitive inflammatory pathways, including nuclear factor kappa B (NF-κB), which amplifies inflammatory and fibrogenic signalling [12,15,17]. NF-κB activation increases the expression of cytokines such as tumour necrosis factor alpha (TNF-α), interleukin-1 beta (IL-1β), and interleukin-6 (IL-6), which can exacerbate renal inflammation and glomerular injury [12,15]. In spontaneously hypertensive rats, chronic NF-κB blockade with pyrrolidine dithiocarbamate reduced blood pressure, lowered cytosolic and mitochondrial oxidative stress, and attenuated renal injury, supporting a mechanistic link between NF-κB and oxidative kidney damage [17]. ROS also promote profibrotic programmes, including transforming growth factor beta (TGF-β) signalling, leading to fibroblast activation, collagen synthesis, and extracellular matrix (ECM) deposition in glomeruli and the interstitium [13]. These processes contribute to fibrosis, podocyte loss, and progressive nephrosclerosis—hallmarks of chronic hypertensive kidney disease. Renin–angiotensin–aldosterone system (RAAS) overactivation, particularly excess angiotensin II (Ang II), can further amplify oxidative stress; in podocytes, Ang II signalling has been linked to increased NOX4 activity and apoptosis, contributing to proteinuria [15,16]. Mitochondria represent an additional ROS source: disruption of the respiratory chain and reduced activity of antioxidant systems (e.g., superoxide dismutase 2 [SOD2]– and sirtuin 3 [SIRT3]–linked defences) promote oxidative injury and bioenergetic failure in tubular epithelial cells [13,18]. Consequently, a loss of mitochondrial membrane potential, reduced adenosine triphosphate (ATP) generation, and increased cell death can aggravate tubular damage and accelerate functional decline [18]. Collectively, these mechanisms create a self-perpetuating cycle in which ROS activates RAAS and inflammatory signalling, which in turn further increases ROS generation and fibrotic remodelling [17]. Therapeutic strategies that blunt RAAS activity (angiotensin-converting enzyme inhibitors/angiotensin receptor blockers), block mineralocorticoid receptors (MRs), or modulate metabolic–haemodynamic stress (e.g., sodium–glucose cotransporter 2 inhibitors) have been associated with reduced oxidative stress and slower kidney disease progression, although direct antioxidant supplementation has shown mixed results across studies [12,15,17]. Overall, oxidative stress integrates haemodynamic, inflammatory, and metabolic disturbances in hypertensive kidney injury and remains an important target for renoprotective intervention [12,13,14,15].

### 2.3. Interstitial Fibrosis

Interstitial fibrosis is a final common pathway of many types of CKD, including HN, and is strongly associated with a progressive loss of renal function [12]. It is characterised by excessive ECM deposition—predominantly fibrillar collagens (types I and III) and basement membrane components (e.g., type IV collagen)—which disrupts tissue architecture and impairs filtration and tubular function [19]. In hypertension, interstitial fibrosis arises from chronic mechanical stress, sustained fibroblast activation, and tubular epithelial injury [4]. TGF-β1 is a central fibrogenic mediator that promotes fibroblast-to-myofibroblast differentiation and induces expression of collagen- and fibronectin-encoding genes [20]. TGF-β1 can also induce epithelial–mesenchymal transition (EMT)-like transcriptional programmes in the tubular epithelium, contributing to a profibrotic tubular phenotype and paracrine activation of interstitial cells, although the extent of complete epithelial-to-mesenchymal conversion in vivo remains model- and context-dependent [4,20]. In unilateral ureteral obstruction (UUO) models, blockade of TGF-β signalling reduces the severity of interstitial fibrosis, supporting the causal role of this pathway in fibrogenesis [20]. Lineage-tracing studies indicate that a large fraction of renal myofibroblasts originates from resident stromal cells (including fibroblasts/pericytes), with additional contributions from bone marrow-derived cells and endothelial transdifferentiation reported in some settings [21]. Hypertensive kidney injury is accompanied by oxidative stress and NF-κB activation, which can increase expression of TGF-β1 and pro-inflammatory cytokines (e.g., TNF-α, IL-6), thereby coupling inflammation to fibrogenesis. ROS and NF-κB can form a positive feedback loop that sustains inflammatory–fibrotic signalling and accelerates interstitial scarring [12,22]. Interstitial hypoxia is another key driver of fibrosis and can arise from peritubular capillary loss, vasoconstriction, and increased diffusion distance due to matrix expansion—features relevant to chronic hypertension. Hypoxia stabilises hypoxia-inducible factor 1 alpha (HIF-1α) and hypoxia-inducible factor 2 alpha (HIF-2α), which can activate profibrotic transcriptional programmes and remodel microvascular responses; however, downstream effects (including vascular endothelial growth factor [VEGF] signalling) are context-dependent [23]. The ‘chronic hypoxia hypothesis’ posits that hypoxia is not merely a consequence of fibrosis but also an active promoter of fibrogenesis in CKD [23,24]. RAAS activation also contributes to fibrotic progression by stimulating oxidative stress and upregulating TGF-β1 in tubular epithelial cells [4]. Ang II signalling via Ang II type 1 receptors can induce fibroblast proliferation, myofibroblast differentiation, and enhanced collagen synthesis, thereby promoting interstitial fibrosis [12,24]. Mitochondrial dysfunction in tubular epithelial cells may further promote fibrosis by impairing ATP production and increasing ROS generation, which amplifies TGF-β1 signalling and epithelial cell loss [18]. Morphologically, interstitial fibrosis is accompanied by tubular atrophy, collagen accumulation, and increased tissue stiffness, which can further impair perfusion and worsen hypoxia [24]. In biopsy series of malignant hypertension, extensive interstitial fibrosis and nephrosclerosis have been reported alongside microangiopathic changes, correlating with severe clinical presentation [11]. Taken together, interstitial fibrosis in hypertension reflects coordinated haemodynamic, oxidative, inflammatory, and metabolic injury pathways, and targeting key nodes such as TGF-β1, NF-κB, RAAS, and hypoxia signalling remains a major therapeutic focus [12,18,22,24].

## 3. Key Molecular Pathways Involved in Disease Progression

### 3.1. RAAS

Chronic RAAS activation contributes to hypertensive kidney disease not only by increasing intrarenal and systemic pressure but also by initiating cell-signalling programmes that promote inflammation and scarring. Ang II can directly activate intracellular pathways in vascular and renal cells, shifting the balance towards ECM accumulation and structural remodelling. A key mechanism linking Ang II to tissue injury is the induction of oxidative stress via redox enzymes, particularly NADPH oxidases, which increases local ROS production and amplifies injury signalling [25]. In experimental settings, Ang II signalling can elicit renal inflammatory injury and fibrotic remodelling through innate immune-related mechanisms, supporting the concept that RAAS can drive fibrosis beyond haemodynamic load alone [26]. Within the RAAS axis, aldosterone acting through the MR is increasingly recognised as a pro-inflammatory and profibrotic mediator that can exacerbate structural kidney damage [27]. Outcome data for finerenone, a non-steroidal MR antagonist, support the clinical relevance of this pathway by showing reduced kidney-related adverse outcomes in CKD with type 2 diabetes [27,28].

### 3.2. TGF-β Pathway

Fibrotic progression in the kidney is strongly orchestrated by TGF-β signalling, which promotes ECM synthesis and the persistence of activated profibrotic cellular states. Canonical TGF-β/Smad signalling is frequently described as a central route through which diverse upstream stressors are translated into sustained myofibroblast activity and interstitial matrix expansion. In the tubular epithelium, TGF-β can induce EMT-like responses associated with fibrogenesis, although the extent of complete EMT in vivo remains context-dependent [29]. Oxidative stress can interact with TGF-β signalling, strengthening profibrotic gene expression and reinforcing progression of renal scarring [30]. Thus, renal fibrosis can be conceptualised as a network in which TGF-β provides a core profibrotic engine, while redox imbalance modulates both the magnitude and durability of these outputs [29,30].

### 3.3. Inflammatory and Immune Pathways

Inflammation is not merely a correlate of hypertensive renal injury but can act as an amplifier that accelerates structural damage and functional decline. IL-6 has been implicated as a contributor in salt-sensitive hypertension models, where pharmacological inhibition of IL-6 signalling attenuated both hypertension and renal injury [31]. Renal macrophage accumulation is a recurring finding in hypertensive kidney injury, and chemokine-driven recruitment pathways such as monocyte chemoattractant protein-1 are associated with inflammatory infiltration and injury severity. These immune cell-rich lesions provide a context in which cytokines and oxidative stress mutually reinforce one another and favour progression towards fibrosis [32]. Complement activation has also been proposed as an immune effector relevant to severe hypertensive phenotypes, including malignant hypertension [33]. Observations of sustained alternative complement pathway activation in malignant hypertension further support a model in which innate immune cascades may participate in ongoing vascular and renal injury [34].

### 3.4. Oxidative Stress Pathways (Nrf2, NOX Enzymes)

Oxidative stress represents a convergent mechanism in hypertensive kidney injury, integrating hormonal, vascular, and immune signals into redox-driven tissue remodelling [15]. Among enzymatic sources of ROS, renal NADPH oxidases are repeatedly emphasised as major contributors shaping both functional dysregulation and fibrotic progression [35,36]. NOX-dependent ROS generation modulates signalling pathways that promote inflammation, endothelial dysfunction, and fibrogenesis in hypertension [15,37]. In the kidney, NOX isoforms have been linked to phenotypes across multiple renal pathologies, consistent with the concept that redox enzymes can act as chronic ‘signal amplifiers’ rather than transient stress responders [37,38]. The role of NADPH oxidase activity is context-dependent and can influence whether cells adapt or undergo injury, underscoring why persistent activation in hypertension is particularly detrimental [39]. Conversely, nuclear factor erythroid 2–related factor 2 (Nrf2) regulates antioxidant defences, and targeting Nrf2 has been discussed as a strategy to reduce oxidative stress and inflammation in CKD [40]. Nrf2 signalling may be impaired in severe hypertensive states, with evidence of downregulation of Nrf2 and associated antioxidant genes in hypertension, consistent with an ‘antioxidant response failure’ phenotype [41].

### 3.5. Wnt/β-Catenin and Notch Signalling

Developmental pathways such as Wnt and Notch can be reactivated after injury and have been proposed as regulators of fibrogenic remodelling in adult kidneys. Rather than operating in isolation, Wnt and Notch intersect with inflammatory and profibrotic circuits, potentially stabilising maladaptive repair programmes and sustaining chronic scarring [42]. Notch activation has been linked to renal fibrogenesis, with evidence that epithelial Notch signalling can drive tubulointerstitial fibrosis in experimental models and human disease contexts [43]. Accordingly, the Notch pathway has been framed as a plausible therapeutic target in CKD given its capacity to shape fibrosis-related cellular states [44]. Together, Wnt/β-catenin and Notch pathways extend classic RAAS–oxidative stress–inflammation models by providing mechanisms through which transient hypertensive insults may be converted into durable profibrotic transcriptional programmes [42,44].

## 4. Characterisation of ncRNAs

ncRNAs are a diverse group of RNA molecules whose main characteristic is the lack of protein-coding capacity, allowing them to play important roles in regulating gene expression. They participate in the regulation of numerous cellular processes through complex mechanisms. ncRNAs can be broadly divided into two categories: housekeeping and regulatory. The former include transfer RNAs (tRNAs) and ribosomal RNAs (rRNAs), which support basic cellular functions. Regulatory RNAs, in turn, modulate gene expression and include linear RNAs—long (lncRNAs) and small (miRNAs, small interfering RNAs [siRNAs], and Piwi-interacting RNAs [piRNAs])—as well as circRNAs. The best-studied ncRNAs are miRNAs, lncRNAs, and circRNAs [45].

miRNAs are small, single-stranded molecules containing 20–24 nucleotides. Their primary role is the regulation of gene expression at the post-transcriptional level [46]. miRNAs bind to target messenger RNA (mRNA) molecules via complementary sequences located in the 3′ untranslated region (3′ UTR). This interaction promotes mRNA degradation or inhibits translation of target genes [47]. Because a single miRNA can target multiple mRNAs, these molecules can influence a wide range of cellular regulatory networks. In addition, miRNAs show a high degree of evolutionary conservation across species and often exhibit tissue-specific expression profiles, suggesting specialised and precise regulatory functions [48].

lncRNAs are heterogeneous transcripts longer than 200 nucleotides. Depending on their cellular localisation, they perform diverse functions. lncRNAs can be found in both the nucleus and the cytoplasm [49]. Within the nucleus, lncRNAs can bind to specific regions of the genome and influence the recruitment of chromatin-modifying complexes as well as transcription factors. In this way, they affect chromatin organisation, epigenetic regulation, and gene transcription. When located in the cytoplasm, lncRNAs can interact with mRNAs and miRNAs. In these contexts, lncRNAs may act as molecular sponges that sequester miRNAs or bind directly to mRNAs, thereby influencing splicing control, stability, and translation [50,51].

circRNAs are a class of lncRNAs that form covalently closed loop structures, which makes them naturally resistant to exonucleases and therefore relatively stable in the cytoplasm [45]. circRNAs are generated through non-canonical splicing, known as back-splicing, in which the 5′ and 3′ ends of an RNA transcript are joined to form a stable circular structure [52]. They may originate from exons, introns, UTRs, or intergenic regions of the genome. Accordingly, circRNAs can be categorised based on their sequence composition: exonic circRNAs, circular intronic RNAs, and exon–intron circRNAs [45]. circRNAs perform diverse regulatory functions within the cell. They participate in gene regulation through the competitive endogenous RNA (ceRNA) mechanism by sequestering miRNAs and acting as molecular sponges, thereby regulating the availability of miRNAs for their target mRNAs. In addition, circRNAs can directly bind to mRNA molecules, influencing the regulation of their function [53]. circRNAs may also interact with proteins, including RNA-binding proteins, thereby modulating their activity in various cellular processes [54].

## 5. The Role of microRNA in the Pathogenesis of HN

### 5.1. Association of miRNAs with Vascular Damage and Endothelial Dysfunction

In AH, endothelial dysfunction is one of the key factors leading to gradual damage to renal vessels. A growing body of evidence indicates that miRNAs play an important role in this process by regulating endothelial cell function, mainly through their influence on the NO pathway and the expression of eNOS [55,56]. The best-known miRNAs associated with this mechanism include miR-155, miR-214-3p, miR-24, and miR-221/222, which interact with the NOS3 gene and related signalling pathways responsible for maintaining normal NO bioavailability.

**Figure 1:**
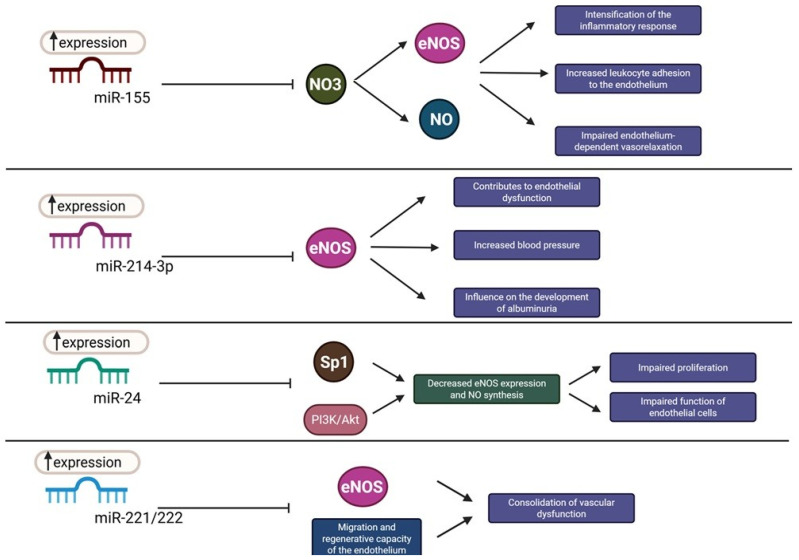
The role of selected microRNAs in the regulation of the eNOS/NO axis and endothelial dysfunction in hypertension. Overexpression of miR-155, miR-214-3p, miR-24, and miR-221/222 leads to inhibition of eNOS expression or activity through direct or indirect molecular mechanisms, resulting in reduced NO production, increased inflammation, endothelial dysfunction, and progression of hypertension-related vascular changes. Explanation: ↑ increased expression Created in BioRender. Plewa, P. (2026) https://BioRender.com/c0dgkkt accessed on 6 March 2026.

miR-155 is one of the best-characterised miRNAs involved in the regulation of endothelial function. Its overexpression leads to direct inhibition of the NOS3 transcript, resulting in reduced eNOS levels and decreased NO production. As a consequence, inflammatory responses are intensified, leukocyte adhesion to the endothelium increases, and endothelium-dependent vasorelaxation is impaired (Figure 1) [55]. Similar effects are observed with miR-214-3p, whose expression increases in hypertension. This miRNA limits eNOS expression in renal vessels, contributing to endothelial dysfunction, increased blood pressure, and the development of albuminuria [57]. Experimental studies have shown that inhibition of miR-214-3p in the kidneys improves NO-dependent signalling and exerts a nephroprotective effect, highlighting its pathogenic significance in AH (Figure 1) [56,58].

miR-24 also affects the eNOS/NO axis, although this mechanism is indirect. This miRNA inhibits the activity of the specificity protein 1 transcription factor and the phosphoinositide 3-kinase/protein kinase B (PI3K/Akt) pathway, which secondarily leads to decreased eNOS expression and reduced NO synthesis. These disturbances translate into impaired proliferation and function of endothelial cells (Figure 1) [59]. By contrast, miR-221/222, whose expression increases under conditions of chronic haemodynamic stress, not only limit eNOS activity but also inhibit endothelial migration and regenerative capacity, thereby contributing to persistent vascular dysfunction (Figure 1) [60].

In summary, dysregulation of selected miRNAs in hypertension leads to a sustained reduction in NO bioavailability, increased oxidative stress, and pro-inflammatory activation of the endothelium. These mechanisms play an important role in the development and progression of HN, highlighting the importance of miRNAs as potential biomarkers and therapeutic targets in hypertensive disease.

### 5.2. miRNAs Modulating the Inflammatory Response

In AH, dysregulation of miRNA expression may promote inflammatory responses. Increased albuminuria has been shown to correlate with elevated levels of miR-29b, which in turn are positively associated with inflammatory markers such as C-reactive protein and TGF-β1 [61]. Experimental studies in mouse and cell models have also confirmed the pro-inflammatory role of specific miRNAs in hypertension. Yang’s group demonstrated that increased endothelial expression of miR-505 induced by hypertension significantly increases the production of IL-1β and TNF-α and promotes monocyte adhesion, thereby exacerbating endothelial dysfunction and vascular inflammation [62]. By contrast, in patients with resistant AH, a significant increase in the serum miR-21 concentration was observed, whereas miR-155 levels remained unchanged [63]. Moreover, miR-21 correlated positively with high aldosterone levels, blood pressure, and other risk parameters, suggesting its involvement in the accumulation of inflammatory mediators. Disturbances in these miRNAs therefore contribute to excessive activation of inflammatory pathways, aggravating endothelial injury and promoting HN. These findings indicate potential diagnostic and therapeutic value in modulating miRNA expression as a means of controlling hypertensive kidney inflammation. Additional miRNAs may also contribute to inflammatory and injury-related pathways in HN. miR-103a-3p has been reported to be increased in patients with HN and in experimental Ang II-induced renal injury, where it was associated with activation of the SNRK/NF-κB/p65 axis, enhanced renal inflammation, and progression of fibrosis. miR-429 has also been linked to hypertensive nephrosclerosis, with increased intrarenal expression correlating with disease severity. These observations suggest that selected miRNAs may contribute not only to systemic vascular inflammation, but also to intrarenal inflammatory and injury signalling in hypertension [64].

### 5.3. miRNAs Regulating Renal Fibrosis (e.g., TGF-β/SMAD)

HN is associated with progressive fibrosis of the interstitium and glomeruli. The key profibrotic factor TGF-β, which activates SMAD family member 3 (Smad3), stimulates the expression of profibrotic miRNAs such as miR-21 and let-7i-5p while simultaneously reducing the expression of antifibrotic miRNAs, including members of the miR-29 family [65,66]. Recent studies indicate that modulation of these miRNAs can influence the progression of fibrosis. In a UUO mouse model, let-7i-5p expression was strongly induced by TGF-β/Smad3 signalling. Using the clustered regularly interspaced short palindromic repeats/CRISPR-associated protein 13d (CRISPR/Cas13d) system, silencing of let-7i-5p significantly reduced fibrotic features by lowering the expression of α-smooth muscle actin (α-SMA), fibronectin, and collagen I [67]. Binding of Smad3 to the let-7i promoter was also confirmed, supporting the dependence of its expression on the TGF-β/Smad3 pathway. Similarly, studies using a rat model showed that overexpression of miR-29b significantly reduced both TGF-β1 levels and the expression of genes associated with EMT and ECM accumulation, including collagen and α-SMA [68]. Conversely, inhibition of miR-29b exacerbated fibrotic changes. The antifibrotic effect of miR-29b appears to involve direct suppression of TGF-β signalling and inhibition of excessive collagen production.

### 5.4. Changes in the miRNA Profile in Serum and Urine of Patients with Hypertension

The miRNA profile in systemic fluids of individuals with hypertension differs significantly from that observed in healthy individuals, highlighting their potential as biomarkers. Clinical studies show that patients with hypertension exhibit characteristic changes in circulating miRNA concentrations. Kara et al. confirmed that in patients with resistant hypertension, the level of miR-21 in serum was significantly higher than in healthy individuals [63]. hsa-miR-184, hsa-miR-432-5p, and hsa-miR-1-3p, whose expression was significantly reduced, as well as hsa-miR-1246, whose expression was increased, have also been identified as potential biomarkers. These miRNAs were found to correlate with the presence of hypertension independently of the patient’s body mass index [69]. In addition, characteristic miRNAs have been detected in the serum and urine of patients with HN and albuminuria. Perez-Hernandez et al. described a group of 29 dysregulated circulating miRNAs associated with albuminuria, four of which were confirmed in serum and urine exosomes. In particular, miR-26a, a regulator of the TGF-β pathway, was significantly reduced in the exosomal fraction of both plasma and urine in patients with albuminuria compared with controls and hypertensive patients without albuminuria [70]. These findings suggest that measuring selected miRNAs in serum and urine may provide a non-invasive method for monitoring the progression of HN and detecting early renal dysfunction.

## 6. The Role of lncRNAs in HN

In recent years, the role of lncRNAs has emerged as a critical regulatory layer in the pathogenesis of HN. lncRNAs function through diverse mechanisms, such as acting as molecular sponges for miRNAs, scaffolding proteins for chromatin-modifying complexes, and modulating gene expression at transcriptional, post-transcriptional, and epigenetic levels [71,72,73]. Dysregulated lncRNAs have been implicated in various renal diseases, including DN and acute kidney injury, and their specific contributions to HN are increasingly recognised through genome-wide studies and animal models [74,75]. This section discusses the roles of lncRNAs in key processes involved in HN, including the regulation of angiogenesis, the modulation of oxidative stress, control of apoptosis, renal remodelling, and fibrosis formation. It also highlights specific lncRNAs such as metastasis-associated lung adenocarcinoma transcript 1 (MALAT1), H19, and plasmacytoma variant translocation 1 (PVT1), drawing on recent studies to underscore their pathogenic significance and potential as therapeutic targets.

### 6.1. The Role of lncRNAs in Regulating Angiogenesis, Oxidative Stress, and Apoptosis

Angiogenesis is essential for maintaining renal tissue oxygenation but becomes dysregulated in HN, leading to peritubular capillary rarefaction, hypoxia, and subsequent tissue damage [76,77]. Under hypertensive conditions, elevated levels of Ang II and aldosterone disrupt endothelial cell homeostasis, impairing angiogenic signalling pathways such as VEGF and HIF-1α [78,79]. lncRNAs serve as important regulators in this context, sometimes referred to as ‘Angio-LncRs’, by influencing endothelial cell proliferation, migration, and tube formation [76,80,81]. For example, pro-angiogenic lncRNAs enhance VEGF expression, whereas anti-angiogenic lncRNAs suppress it, creating a delicate balance that may be disrupted in HN. Oxidative stress, another hallmark of HN, arises from an imbalance between ROS production and antioxidant defences, primarily driven by mitochondrial dysfunction and NADPH oxidase activation in renal cells [1,82]. This stress amplifies endothelial injury and promotes apoptosis, the programmed cell death that leads to podocyte and tubular cell loss and further exacerbates renal decline [71,83]. lncRNAs integrate these processes by modulating redox-sensitive pathways, such as nuclear factor erythroid 2–related factor 2/heme oxygenase-1 (Nrf2/HO-1) signalling, and apoptotic cascades involving B-cell lymphoma 2 (Bcl-2) family proteins and caspases [73,84].

**Figure 2:**
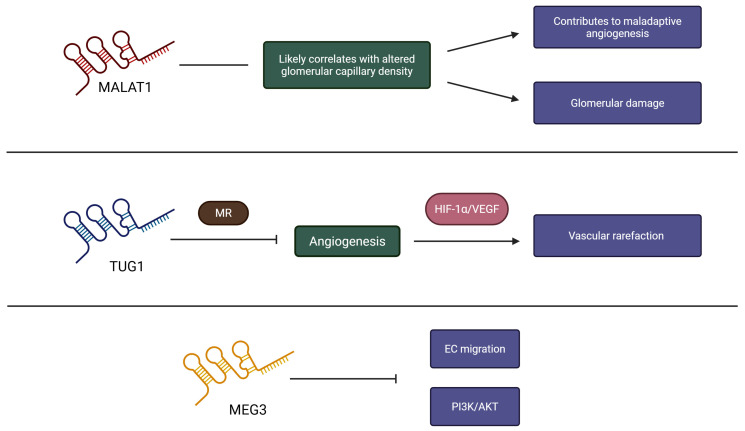
The role of lncRNAs in modulating renal angiogenesis in hypertension. The lncRNA MALAT1 may influence glomerular capillary density, while TUG1 inhibits angiogenesis by attenuating mineralocorticoid receptor-mediated HIF-1α/VEGF signalling. MEG3, in turn, limits endothelial cell migration by regulating miR-21 and inhibiting the PI3K/AKT pathway, which may promote hypoxia and progression of renal injury. Created in BioRender. Plewa, P. (2026) https://BioRender.com/kgvcdm6 accessed on 6 March 2026.

In angiogenesis, lncRNAs such as MALAT1 have been shown to promote endothelial cell function by sponging miR-205-5p, thereby upregulating VEGFA and facilitating vessel sprouting, primarily in models of vascular remodelling associated with hypertension (e.g., Ang II–induced vascular smooth muscle cell [VSMC] proliferation) and in DN or hypoxia models [1,76]. Although direct evidence in pure HN remains limited, studies in rat models of hypertension suggest that MALAT1 may correlate with altered glomerular capillary density, potentially contributing to maladaptive angiogenesis and glomerular injury by analogy with related renal pathologies [75]. Conversely, lncRNA taurine upregulated gene 1 (TUG1) interacts with MRs to inhibit angiogenesis by downregulating HIF-1α/VEGF signalling, resulting in vascular rarefaction observed in UUO models that mimic aspects of HN [78,79]. Anti-angiogenic lncRNAs such as maternally expressed gene 3 (MEG3) suppress endothelial cell migration by targeting miR-21 and inhibiting the PI3K/Akt pathway, and their upregulation in HN may exacerbate hypoxia (Figure 2) [76,82,85]. These mechanisms are further complicated by interactions with other ncRNAs; for instance, ceRNA networks involving lncRNAs and miRNAs can amplify angiogenic dysregulation in HN [75].

Regulation of oxidative stress by lncRNAs is equally important. lncRNA H19 has been implicated in exacerbating ROS production primarily in DN and other fibrotic renal models by modulating the H19/miR-106a axis, which suppresses antioxidant enzymes such as SOD2 and promotes mitochondrial fission [71,73]. Although its role in HN is less directly established, H19 knockdown in endothelial cells exposed to stressors relevant to hypertension reduces NADPH oxidase activity and lipid peroxidation, suggesting a potential pro-oxidative contribution to hypertension-associated renal injury [82]. Similarly, PVT1 enhances oxidative damage through the PVT1/miR-150-5p/angiopoietin-like 4 (ANGPTL4) pathway, leading to mitochondrial ROS accumulation and endothelial dysfunction in HN [85,86]. Apoptosis is closely linked with these processes. MALAT1 can protect against endothelial cell apoptosis by upregulating Bcl-2 and inhibiting caspase-3; however, in HN its dysregulation may shift the balance towards increased cell death [81,87]. By contrast, TUG1 promotes tubular cell apoptosis through the TUG1/miR-542-3p/HIF-1α/VEGF axis, linking oxidative stress to programmed cell death and vascular impairment [79,88]. Therapeutic interventions such as mitochondrial-targeted antioxidants, including MitoQ, have shown promise in mitigating lncRNA-mediated oxidative stress by restoring mitochondrial function and reducing apoptosis in hypertensive models [82]. Together, these findings suggest that targeting lncRNA-regulated networks could alleviate the interconnected processes of angiogenesis dysregulation, oxidative stress, and apoptosis, potentially slowing the progression of HN.

### 6.2. lncRNAs Influencing Renal Remodelling and Fibrosis Formation

Renal remodelling in HN encompasses adaptive and maladaptive changes, including glomerular hypertrophy, arteriolar thickening, and tubular atrophy, driven by haemodynamic stress and inflammatory signals [73,77]. This remodelling often culminates in fibrosis, characterised by ECM deposition by activated myofibroblasts, leading to scar formation and progressive loss of renal function [85,87]. lncRNAs orchestrate these processes by regulating EMT, myofibroblast activation, and ECM synthesis through key pathways such as TGF-β/Smad3 signalling and RAAS activation [78,83]. In renal remodelling, lncRNAs such as TUG1 bind to MRs, enhancing Ang II-induced VSMC proliferation and ECM remodelling, which contributes to arteriolar hypertension and glomerular injury in HN [75,78]. Genome-wide analyses in rat models have identified differentially expressed lncRNAs that correlate with remodelling markers such as collagen I and α-SMA in hypertensive compared with normotensive strains [74,89]. Fibrosis is further amplified by lncRNAs that modulate inflammatory and fibrogenic responses. For example, nuclear paraspeckle assembly transcript 1 (NEAT1) recruits chromatin-modifying complexes to promote IL-6 expression, thereby fostering macrophage infiltration and interstitial fibrosis in HN [71,84]. By contrast, lncRNA TGF-β/Smad3-interacting lncRNA (lnc-TSI), a kidney-enriched lncRNA, inhibits TGF-β/Smad3 phosphorylation and attenuates EMT and fibrotic progression; its downregulation in hypertensive kidneys may therefore accelerate renal remodelling [87]. Epigenetic mechanisms, including DNA methylation and histone acetylation influenced by lncRNAs, further contribute to these changes. MALAT1, for example, enhances Smad3 activity through chromatin looping, promoting collagen deposition and tubular atrophy [1,88].

The fibrotic niche in HN involves complex interactions among different renal cell types, with lncRNAs regulating the phenotypes of podocytes, endothelial cells, and tubular epithelial cells [77]. Single-cell RNA sequencing studies have highlighted lncRNA-mediated cellular crosstalk, including disruption of insulin signalling pathways through small nucleolar RNA host gene 14 (SNHG14) within ceRNA networks [75]. In UUO models, fluorescence microangiography has revealed lncRNA-associated peritubular capillary loss, linking renal remodelling to fibrotic progression. Therapeutic targeting of these lncRNAs—for example, through siRNA-mediated knockdown—has attenuated fibrosis in animal models, suggesting potential benefits of combining RAAS inhibition with lncRNA-targeted strategies [79,80,86]. Overall, lncRNAs form an important regulatory hub in HN-associated remodelling and fibrosis, integrating haemodynamic and molecular signals that drive disease progression.

### 6.3. Examples of Investigated lncRNAs Associated with Pathogenesis

A critical issue in interpreting the role of lncRNAs in HN is the heterogeneity of the available evidence. To avoid overinterpretation, it is useful to distinguish three levels of evidence:

(1) Direct HN-specific evidence derived from dedicated hypertensive renal injury models or human HN cohorts;

(2) Evidence from hypertension-associated or mixed cardio-renal injury settings;

(3) Extrapolated evidence from related renal disease models such as diabetic DN, UUO, or general renal fibrosis.

At present, only a limited subset of ncRNA findings can be considered directly validated in pure HN. By contrast, many lncRNAs frequently discussed in the renal fibrosis literature, including MALAT1, H19, PVT1, TUG1, and NEAT1, have been primarily characterised in DN, UUO, or broader CKD contexts. These studies remain biologically informative because they involve pathways highly relevant to HN, such as TGF-β/Smad signalling, oxidative stress, endothelial dysfunction, extracellular matrix deposition, and inflammatory activation. However, extrapolation from these models should be interpreted cautiously, particularly because metabolic abnormalities, hyperglycaemia, and disease-specific microenvironmental factors may substantially influence ncRNA expression and function.

Accordingly, in the context of HN, these lncRNAs should currently be viewed as mechanistically plausible and translationally interesting candidates rather than definitively established HN-specific regulators. Future studies should prioritise validation in dedicated hypertensive kidney injury models and in clinically well-phenotyped human cohorts without confounding diabetes.

Several lncRNAs have been investigated in the context of renal diseases associated with hypertension, although most functional and mechanistic data derive from DN, obstructive nephropathy, or general models of renal fibrosis and CKD. Their specific contributions to pure HN (without concurrent diabetes) require further investigation in dedicated experimental models, such as spontaneously hypertensive rats or Ang II infusion models without hyperglycaemia. MALAT1 is a highly conserved lncRNA strongly associated with vascular remodelling in hypertension models (e.g., Ang II–induced VSMC changes) and is upregulated in various renal fibrotic conditions. It acts as a ceRNA, for example by sponging miR-143-3p to enhance oxidative stress, apoptosis, and angiogenesis through VEGFA-related pathways. In datasets related to hypertension-associated renal injury, MALAT1 participates in ceRNA networks regulating fibrosis markers, and its involvement in TGF-β signalling may promote EMT. In relevant rat models, MALAT1 knockdown has been shown to reduce glomerular injury. Urinary MALAT1 levels have also been proposed as potential biomarkers in fibrotic renal diseases, including those associated with hypertension. MALAT1 is one of the best-characterised lncRNAs in renal fibrosis biology. Nevertheless, in the context of HN, much of the available mechanistic evidence remains indirect and derives from obstructive or metabolic kidney injury models rather than dedicated pure hypertensive nephropathy systems [1,71,75,76,84]. H19, an imprinted lncRNA, is prominently involved in oxidative and fibrotic pathways primarily in DN and other CKD models. It modulates insulin-like growth factor 2 signalling, which can exacerbate mitochondrial ROS production and apoptosis in podocytes. By analogy, H19/miR-106a interactions may contribute to EMT and ECM deposition in hypertension-related renal injury, with overexpression potentially linked to tubular fibrosis. Animal studies demonstrate that H19 silencing ameliorates vascular remodelling and fibrosis in experimental fibrotic models, suggesting potential therapeutic relevance for HN. However, direct HN-specific validation remains limited, and current interpretation should therefore remain cautious. [71,73,74,80,83]. PVT1 contributes to disease pathogenesis mainly in DN, where it sponges miR-150-5p to upregulate ANGPTL4, thereby enhancing oxidative stress and fibrosis. In models combining diabetic and hypertensive conditions, PVT1 promotes podocyte apoptosis and glomerular hypertrophy. Its expression in renal biopsy samples correlates with disease progression in CKD, including cases associated with hypertension, indicating potential utility as a biomarker. PVT1 has repeatedly been associated with fibrosis-related signalling and extracellular matrix accumulation in renal disease. Its biological relevance to HN is plausible, but current evidence is largely extrapolative rather than disease-specific [72,83,85,86]. Other lncRNAs, such as TUG1, interact with MRs to induce Ang II-mediated fibrosis and apoptosis, while SNHG14 regulates insulin signalling pathways within ceRNA networks. In addition to SNHG14, other members of the SNHG family, particularly SNHG12, have been implicated in vascular inflammation, endothelial dysfunction, and renal fibrotic signalling in related disease settings. Although these observations increase the biological plausibility of SNHG family involvement in HN, direct validation in dedicated hypertensive nephropathy models remains limited. Additional transcriptomic lncRNA candidates reported in hypertension-related renal injury include AK094457, AK098656, and MRAK04835_PI. However, the available evidence for these transcripts remains largely discovery-based and insufficiently validated at functional or clinical levels. [75,78,79]. These examples illustrate the multifaceted roles of lncRNAs in renal pathophysiology and highlight their potential as targets for therapeutic intervention.

In conclusion, lncRNAs play central roles in renal pathogenesis associated with hypertension and represent promising candidates for diagnostic and therapeutic development. However, although lncRNAs such as MALAT1, H19, and PVT1 are well characterised in DN and related fibrotic conditions, their specific mechanistic contributions to pure HN require further validation in dedicated experimental models and in human cohorts without confounding diabetes.

## 7. circRNAs as Regulators of Hypertension-Induced Kidney Damage

circRNAs are covalently closed lncRNAs that are relatively resistant to exonuclease degradation. Consequently, they tend to be stable transcripts in cells, including in the kidney. These molecules are capable of regulating gene expression through multiple mechanisms, most commonly by acting as ceRNAs that bind miRNAs. In addition, circRNAs can interact with RNA-binding proteins and modulate transcriptional programmes [45]. In a deoxycorticosterone acetate (DOCA)–salt-induced salt-sensitive hypertension model, kidney RNA sequencing profiling identified thousands of circRNA candidates and a distinct subset of 124 circRNAs whose expression changed in hypertensive renal injury [90,91].

From this set, circNr1h4 (also described as circRNA1350) was selected for further study because it is strongly expressed in kidney tissue and shows marked regulation during DOCA–salt-induced injury. Mechanistic assays demonstrated that circNr1h4 directly associates with miR-155-5p and can function as a molecular ‘sponge’ for this miRNA, as shown by capture and luciferase reporter experiments. The same study confirmed fatty acyl-CoA reductase 1 (Far1) as a direct miR-155-5p target using 3′ UTR reporter assays, thereby defining a circNr1h4 → miR-155-5p → Far1 regulatory axis.

Functionally, knockdown of circNr1h4 or overexpression of miR-155-5p resulted in reduced Far1 expression and increased ROS signals in collecting duct cells. Conversely, overexpression of Far1 attenuated ROS under conditions of lipotoxic stress, establishing a link between this pathway and redox imbalance in hypertensive kidney injury. A recent review of epigenetic mechanisms in salt-sensitive hypertension highlights this circNr1h4/miR-155-5p/Far1 axis as an example of how ncRNA regulation can intersect with oxidative stress-related injury pathways in hypertension [91]. In addition to intrarenal mechanisms, urinary extracellular vesicles (EVs), including exosomes, are being explored as a non-invasive source of kidney-derived molecular signals. circRNAs have therefore been proposed as candidate EV cargo biomarkers in CKD-related remodelling [92,93]. In a Disease Markers study, stimulation of HK-2 tubular epithelial cells with TGF-β1 increased levels of hsa_circ_0008925 in cell-derived exosome samples, supporting a relationship between a profibrotic environment and exosomal circRNA enrichment [94,95]. Clinically, urinary exosomal hsa_circ_0008925 levels were elevated in patients with renal fibrosis and correlated with tubulointerstitial fibrosis and glomerulosclerosis scores [94,95]. The diagnostic performance of this marker for fibrosis was moderate, with an area under the curve of approximately 0.782 and relatively high specificity at the reported cut-off [96]. Another urinary exosome study reported that hsa_circ_0036649 (derived from the A-kinase anchoring protein 13 [AKAP13] locus) correlated with fibrotic pathology scores, including tubulointerstitial fibrosis and glomerulosclerosis, and showed potential diagnostic value. However, the authors emphasised cohort heterogeneity, limited sample size, and the need for mechanistic validation [95]. Consistent with these findings, a review of urinary exosome biomarkers summarised hsa_circ_0008925 and hsa_circ_0036649 as emerging circRNA candidates while emphasising that further validation is required before clinical application [92,93,94,95]. Finally, the therapeutic potential of circRNA stability is also being explored. Engineered circRNAs encoding relaxin-2 have demonstrated antifibrotic efficacy in vivo and showed a favourable safety profile, including no detectable impairment of kidney function in mouse models [96]. Taken together, existing studies support a dual perspective in which circRNAs can act as mechanistic regulators of hypertension-related renal injury—exemplified by the circNr1h4/miR-155-5p/Far1 pathway—while also serving as urinary exosomal indicators of fibrotic remodelling. However, most human urinary circRNA candidates still require causal, hypertension-focused functional validation before their clinical relevance can be fully established [91,92,94,95].

## 8. Interactions Between Different Classes of ncRNAs

### 8.1. miRNA–lncRNA–circRNA Regulatory Networks

Interactions among miRNAs, lncRNAs, and circRNAs form complex regulatory networks. lncRNAs and circRNAs often contain binding sites for specific miRNAs and can act as miRNA sponges, thereby limiting the ability of miRNAs to suppress their target mRNAs. One example is MALAT1, whose expression increases under the influence of TGF-β1. MALAT1 directly binds miR-124-3p, thereby relieving suppression of the integrin beta 1 (ITGB1) gene, a key molecule involved in renal fibrotic processes. Moreover, downregulation of MALAT1 has been shown to reduce the levels of fibrosis markers such as ITGB1, α-SMA, and fibronectin in renal epithelial cells and in the UUO model [97]. Similarly, a study by Kai Ai et al. demonstrated that TGF-β–induced circRNA_33702 binds miR-29b-3p, leading to de-repression of the fibrosis-promoting protein WNT1-inducible signalling pathway protein 1 (WISP1) [98]. Silencing circRNA_33702 attenuated TGF-β1-induced increases in collagen I and III, whereas its overexpression produced the opposite effect. These examples illustrate the influence of miRNA–lncRNA–circRNA regulatory networks on gene expression: modulation of one component of the network can alter the levels of receptors or growth factors involved in pathological processes such as fibrosis and renal inflammation. However, that these examples were primarily characterised in obstructive or general fibrotic kidney injury models rather than in pure HN. Therefore, although they illustrate the biological logic of ceRNA-type interactions in renal disease, their direct relevance to HN should be interpreted with caution.

### 8.2. ceRNA Networks

ceRNA networks represent a regulatory mechanism in which different RNA molecules (mRNAs, lncRNAs, and circRNAs) compete for binding to the same miRNAs. Through this mechanism, one transcript can indirectly influence the expression of another by sequestering shared miRNAs. In the kidney, ceRNA interactions enable precise regulation of genes that are important for maintaining tissue pressure and structural integrity. For example, MALAT1 and circRNA_33702 function as ceRNAs by binding miR-124-3p and miR-29b-3p, respectively, thereby affecting the expression of ITGB1 and WISP1, genes involved in fibrotic pathways [97]. However, these interactions were not validated specifically in HN and should therefore not be regarded as HN-specific ceRNA networks. Bioinformatic analyses have suggested that, in HN-related datasets, the lncRNAs small nucleolar RNA host gene 14 (SNHG14) and TUG1 may act as central hubs within the ceRNA regulatory network [75]. This network appears to regulate components of the insulin signalling pathway through interactions involving protein phosphatase 1 regulatory subunit 3C (PPP1R3C), protein kinase cAMP-dependent type II regulatory subunit beta (PRKAR2B), AKT serine/threonine kinase 3 (AKT3), and miR-107, suggesting a link between ceRNA regulation and metabolic aspects of the disease. Through such competitive interactions, cells can finely tune the expression of proteins involved in blood pressure regulation and vascular homeostasis. Disruption of ceRNA networks, for example through overexpression of specific lncRNAs, may disturb this regulatory balance and lead to pathological expression of profibrotic and pro-inflammatory genes. Notably, the currently available data do not explain how SNHG14- and TUG1-related interactions specifically contribute to hypertensive renal injury, as distinct from diabetic kidney disease or other metabolic forms of kidney damage. Therefore, these networks should currently be interpreted as putative regulatory systems that require further functional validation in dedicated HN models.

One of the most relevant hypertension-associated ceRNA network examples currently available is the circNr1h4/miR-155-5p/Far1 axis identified in the DOCA–salt model of hypertension-induced kidney injury [91]. In this setting, circNr1h4 acts as a molecular sponge for miR-155-5p, thereby preserving the expression of Far1. Dysregulation of this pathway, including circNr1h4 downregulation or miR-155-5p overexpression, leads to reduced Far1 expression and increased ROS generation in collecting duct cells. Functionally, this promotes redox imbalance and tubular injury, linking ceRNA network disruption with oxidative stress-related mechanisms of hypertensive renal damage. Although this axis represents a more disease-relevant example in HN, fully validated lncRNA-centred ceRNA networks specific to pure HN remain limited, which should be recognised as an important area for future research.

### 8.3. The Importance of These Interactions for the Progression of Nephropathy

Interactions among miRNAs, lncRNAs, and circRNAs play an important role in the progression of HN by modulating profibrotic and pro-inflammatory pathways. For example, findings by Xia’s group confirmed that the pathogenic MALAT1/miR-124-3p/ITGB1 network is present not only in UUO models but also in renal tissues from patients with obstructive kidney disease [97]. This observation indicates that increased MALAT1 expression, together with the associated inhibition of miR-124, may exacerbate fibrosis of the renal parenchyma. Similarly, circRNA_33702, through interactions affecting miR-29b-3p signalling, promotes mechanisms leading to collagen accumulation and fibrosis via WISP1-related pathways [98,99]. These findings suggest that dysregulation of ncRNA regulatory networks can directly accelerate the deterioration of renal function in hypertension. From a clinical perspective, changes in miRNA and other ncRNA profiles also have potential as biomarkers of disease progression. One example is miR-26a, whose downregulation has been associated with the onset of albuminuria and an increased risk of nephropathy progression [70]. From a therapeutic standpoint, targeted modulation of these interactions—such as delivery of miR-29b mimics or inhibition of MALAT1 or circRNA_33702—may represent a potential strategy for slowing renal fibrosis in hypertension. Nevertheless, the limited number of ceRNA networks functionally validated in pure HN indicates that further mechanistic studies are still needed before this interaction can be regarded as established therapeutic targets in HN. In summary, miRNA–lncRNA–circRNA regulatory networks influence key molecular processes in HN, with important implications for prognosis and with potential as therapeutic targets in CKD associated with hypertension.

## 9. ncRNAs as Biomarkers and Therapeutic Targets in HN

Conventional diagnostic tools, such as serum creatinine levels, estimated glomerular filtration rate, and the urinary albumin-to-creatinine ratio, have limited sensitivity and often detect disease only after substantial structural damage has occurred. This delay highlights the need for novel biomarkers capable of identifying disease at earlier stages. In this context, ncRNAs—including miRNAs, lncRNAs, and circRNAs—have shown considerable promise. These molecules, detectable in serum and urine, participate in key pathological pathways such as inflammation, fibrosis, endothelial dysfunction, and apoptosis, thereby providing insight into early molecular alterations associated with HN [1,75,89,99,100,101,102,103,104,105,106,107,108].

The diagnostic potential of ncRNAs lies partly in their accessibility through non-invasive biofluid sampling and their ability to reflect dynamic epigenetic changes that precede overt clinical manifestations. In serum, dysregulated miRNAs such as miR-21-5p, miR-126-3p, and miR-192-5p have been associated with the progression of HN. miR-21-5p is upregulated and linked to fibrotic TGF-β/Smad signalling, whereas miR-126-3p is downregulated and correlates with endothelial injury and reduced glomerular filtration [81,103,104]. Similarly, lncRNAs such as SNHG14 and TUG1 have been reported to show elevated serum levels in HN, where they regulate pathways including insulin signalling and cytokine-mediated inflammation. circRNAs, owing to their covalently closed loop structures, exhibit enhanced stability and are frequently enriched in exosomes. For example, circRNA_000203 is upregulated in serum and functions as a molecular sponge for miR-26b-5p, thereby promoting fibrosis in experimental HN models [71,75,86,102,105,106]. In addition, hsa_circ_0014243 and hsa_circ_0037911 have been proposed as circulating biomarkers of essential hypertension. However, their diagnostic value for HN specifically has not yet been established and therefore requires further validation in HN-focused studies [107,108].

Urine-based ncRNAs provide complementary information and may more directly reflect localised renal injury. Urinary miR-192-5p and miR-200b have been reported to show increased excretion in HN and are associated with tubular EMT and podocyte loss [109]. lncRNAs such as MALAT1 are also detectable in urinary exosomes and have been correlated with renal fibrosis through mechanisms involving NF-κB activation. Some studies suggest that urinary ncRNAs may detect subclinical HN before the onset of albuminuria, with area under the curve values reaching approximately 0.92 for combined panels including miR-126 and miR-192. Earlier detection could allow for more timely therapeutic intervention, potentially reducing progression to ESRD through improved blood pressure control [84,100,105,110,111,112].

ncRNAs possess several advantages as potential biomarkers. They demonstrate notable stability in biofluids and are resistant to degradation by RNases, which supports their suitability for routine clinical assays. In addition, their tissue- and disease-specific expression profiles allow for the development of multiplex panels that improve diagnostic accuracy. For example, combined analysis of miR-21, miR-126, and miR-192 has been reported to provide higher sensitivity (0.88–0.91) than individual markers alone. Moreover, ncRNAs reflect upstream regulatory events and can therefore offer mechanistic insights into HN pathogenesis, including miRNA–lncRNA interactions affecting TGF-β-related pathways [46,50,111,112,113,114].

Despite these promising features, several limitations remain. Interindividual variability influenced by age, sex, ethnicity, comorbidities (e.g., diabetes), and medication use may confound results. For example, overlapping ncRNA profiles between HN and DN can complicate differential diagnosis. Furthermore, the relatively low abundance of ncRNAs in biofluids often necessitates highly sensitive analytical techniques, such as droplet digital polymerase chain reaction, which increases assay complexity and cost. Pre-analytical variables—including sample handling, storage conditions, and normalisation methods—may introduce additional bias, and the absence of standardised reference values limits reproducibility. In addition, the pleiotropic nature of miRNAs may lead to off-target effects and reduced specificity because similar regulatory roles are observed in other forms of CKD [101,102,113,115,116,117]. Translational research in this area is progressing. Preclinical studies using models such as Ang II-infused mice have validated several ncRNA signatures associated with hypertensive kidney injury. Analyses of publicly available human datasets (e.g., Gene Expression Omnibus dataset GSE37460) have also identified hub genes, including nuclear receptor subfamily 4 group A member 1 (NR4A1) and C–C motif chemokine ligand 5 (CCL5), that may be regulated by ncRNAs in HN. However, clinical trials remain limited. For instance, miR-21 is currently being evaluated as a biomarker in fibrosis-related diseases, but trials specifically focused on HN are still lacking [113]. Ongoing research increasingly emphasises the potential of exosome-derived ncRNAs for improved specificity, as well as the need for large cohort studies to enable standardisation and validation [72,84,102,114,116]. Future translational efforts are expected to focus on integrating ncRNA signatures into risk stratification algorithms for patients with hypertension. Such approaches could substantially improve early screening strategies and contribute to the development of precision medicine approaches for the management of HN.

Importantly, the clinical usefulness of ncRNAs should be judged not only by statistical significance but also by whether they improve risk prediction beyond established markers such as eGFR and urinary albumin excretion. At present, no ncRNA biomarker has demonstrated sufficient evidence to replace these conventional tools in routine clinical practice. The most realistic near-term application may therefore lie in complementing standard renal assessment by identifying earlier molecular perturbations, stratifying fibrotic or inflammatory endophenotypes, or refining prognostic models in patients with hypertension.

### 9.1. Translational Challenges of ncRNA-Based Biomarkers in Hypertensive Nephropathy

Although ncRNAs are attractive biomarker candidates because of their relative stability and mechanistic relevance, their clinical translation in HN remains limited by several important challenges. First, most available studies are exploratory, based on relatively small cohorts, often conducted at a single centre, and frequently lack external validation. In many cases, reported biomarker performance metrics are incomplete, with limited information on sensitivity, specificity, positive predictive value, negative predictive value, or incremental value over established clinical markers.

Second, disease specificity remains a major concern. Many ncRNAs implicated in HN are also dysregulated in diabetic nephropathy, general CKD, obesity-associated renal injury, and systemic vascular inflammation. This overlap complicates interpretation and raises the possibility that some reported signatures may reflect fibrosis, albuminuria, endothelial dysfunction, or chronic inflammation more broadly rather than HN specifically. In particular, diabetes, insulin resistance, obesity, and antihypertensive treatment may substantially modify ncRNA expression profiles and should be considered major confounders in both mechanistic and clinical studies. Third, pre-analytical and analytical variability remains a substantial barrier to implementation. Important sources of heterogeneity include the biological matrix analysed (serum, plasma, whole urine, urinary sediment, extracellular vesicles, or tissue), centrifugation and storage protocols, haemolysis, freeze–thaw cycles, RNA extraction efficiency, and the choice of endogenous or exogenous normalisation controls. In addition, results may differ significantly depending on the analytical platform used, including qPCR, droplet digital PCR, microarrays, and RNA sequencing. The absence of universally accepted reference standards currently limits reproducibility and inter-study comparability.

Finally, for clinical adoption, ncRNA biomarkers would need to demonstrate value beyond existing tools such as estimated glomerular filtration rate (eGFR), albuminuria, and conventional cardiovascular–renal risk stratification. At present, available evidence does not support replacement of established clinical markers. Rather, ncRNAs may be more realistically viewed as complementary biomarkers with potential value in earlier molecular risk stratification, disease endophenotyping, or prediction of progression, pending robust prospective validation in well-defined HN cohorts [1,71,85,101,102,114,116].

### 9.2. c AntagomiRs, miRNA Mimics, lncRNA Inhibitors, circRNA-Based Therapies, Development Challenges, Clinical Prospects, and Future Directions

Epigenetic modulation via ncRNAs represents a promising therapeutic avenue for HN and may help address the limitations of conventional treatments, such as RAAS inhibitors, which often fail to halt disease progression because they do not fully target fibrotic and inflammatory pathways. Emerging therapeutic strategies include antagomiRs, miRNA mimics, lncRNA inhibitors, and circRNA-based modulators, all of which exploit the regulatory role of ncRNAs in gene expression to mitigate renal injury [1,50,101,116].

antagomiRs are antisense oligonucleotides designed to bind and inhibit pathogenic miRNAs. These agents have shown encouraging biological effects in preclinical models relevant to hypertensive kidney injury or renal fibrosis. For instance, antagomiR-21 suppresses TGF-β/Smad3-driven fibrosis by derepressing antifibrotic target genes, thereby reducing glomerulosclerosis in hypertensive rodent models. Similarly, antagomiR-214 targets proapoptotic pathways and helps preserve podocyte integrity. These molecules are often chemically modified with locked nucleic acids to improve stability and binding affinity. Some antagomiR-based therapies have already progressed to clinical trials for other fibrotic diseases; for example, lademirsen, targeting miR-21, has reached phase II trials for Alport syndrome, supporting the conceptual feasibility of similar approaches in HN, although disease-specific clinical evidence remains unavailable [106,111,115,116]. miRNA mimics represent another therapeutic strategy aimed at restoring the function of protective miRNAs that are downregulated in disease. For example, miR-126 mimics have been shown to enhance vascular repair by targeting the chemokine receptor CX3CR1 and promoting angiogenesis, thereby improving endothelial function in experimental models. Similarly, miR-29 mimics suppress ECM deposition through modulation of Smad signalling and have demonstrated renoprotective effects in hypertensive kidney disease models. These double-stranded RNA analogues are often delivered using nanoparticle-based systems. miR-29 mimics have already entered early clinical testing, including phase I trials for scleroderma, with potential future application in HN [50,103,110,114,116]. lncRNA inhibitors represent another emerging approach. These may include siRNAs, gapmers, or antagoNATs—antisense oligonucleotides designed to target natural antisense transcripts. Experimental studies have shown that inhibition of lncRNAs such as SNHG14 or MALAT1 using siRNA can reduce inflammation and fibrosis by modulating NF-κB and TGF-β signalling pathways in models of hypertensive kidney injury. antagoNAT strategies have also demonstrated the ability to upregulate protective genes such as brain-derived neurotrophic factor (BDNF) in central nervous system models, suggesting potential for adaptation to renal disease. Gene-editing approaches using CRISPR–Cas9 to silence lncRNA loci have also been explored, although challenges related to delivery and safety remain [71,86,116]. circRNA-based therapies take advantage of the miRNA-sponging properties of circRNAs. Synthetic circRNAs designed to mimic protective endogenous circRNAs may sequester pathogenic miRNAs and thereby inhibit downstream fibrotic signalling. For example, engineered circRNA analogues related to circRNA_000203 may bind miR-26b-5p and suppress EMT-associated pathways in HN. Gene-editing approaches using CRISPR–Cas13 have also been proposed as a means of modulating circRNA expression. In preclinical studies, vectors promoting circRNA overexpression have been shown to reduce renal fibrosis in hypertensive mouse models [101,102,112].

Development challenges encompass delivery, safety, and specificity. Efficient renal delivery is hindered by ncRNA instability and glomerular filtration barriers; lipid nanoparticles, exosomes, and renal-targeted conjugates (e.g., anti-podocyte antibodies) can improve uptake but still face endosomal entrapment and off-site accumulation. Safety issues include immunogenicity from unmodified RNAs and hepatotoxicity associated with overdosing, which may be mitigated by chemical modifications such as 2′-O-methyl groups. Specificity is further complicated by miRNA multitargeting, which can lead to unintended effects; strategies such as seed sequence optimisation and tissue-specific promoters may partially address this issue, although redundancy among ncRNAs suggests that combinatorial approaches may be required [46,50,84,114,116,117]. Clinical prospects are nevertheless encouraging, with miRNA therapeutics such as antagomiR-17 currently in phase III trials for polycystic kidney disease, indicating potential applicability to HN. lncRNA inhibitors, including those targeting BDNF-AS, have demonstrated the ability to cross the blood–brain barrier, inspiring renal delivery innovations such as minimally invasive nasal depot approaches [116]. circRNA-based therapies remain at an early stage but benefit from inherent molecular stability, which may support their development in exosome-based delivery systems for HN [84,89].

The table below (Table 1) presents selected ncRNA molecules indicated as potential biomarkers in hypertensive nephropathy and the accompanying renal fibrosis.

Future directions emphasise personalised medicine approaches using multi-omics profiling to identify patient-specific ncRNA targets. Artificial intelligence-based prediction of ncRNA interactions may accelerate drug discovery and design, while phase II/III trials of therapies specifically targeting HN are anticipated. Addressing overlaps between HN and DN through shared molecular pathways (e.g., insulin signalling) may also enable the development of dual-purpose therapeutic strategies [1,71,75,102]. Ultimately, ncRNA-based therapies hold promise for slowing or halting the progression of HN, potentially reducing the global burden of ESRD through targeted epigenetic intervention.

## 10. Conclusions and Future Perspectives

Accumulating experimental and clinical evidence indicates that HN is not solely a consequence of chronic haemodynamic stress but rather a disease with complex molecular pathogenesis in which epigenetic and post-transcriptional regulators, including ncRNAs, play important roles. miRNAs, lncRNAs, and circRNAs form interconnected regulatory networks that modulate renal vascular function, oxidative stress, inflammatory responses, and fibrotic processes, thereby influencing the progression of renal damage in hypertension. Numerous studies have demonstrated their involvement in endothelial dysfunction, enhanced inflammation, and activation of profibrotic pathways, particularly the TGF-β/Smad axis. Dysregulation of miRNAs such as miR-155, miR-21, miR-29, and let-7i-5p has been associated with both the severity of albuminuria and histological markers of interstitial fibrosis. These findings suggest that miRNAs may act as molecular ‘amplifiers’ of haemodynamic signals, sustaining pathological cellular phenotypes even when blood pressure is partially controlled.

In parallel, an increasing number of studies have described lncRNAs that integrate signals derived from RAAS activation, oxidative stress, and inflammatory responses. lncRNAs such as MALAT1, H19, PVT1, and TUG1 modulate angiogenesis, tubular cell survival, and myofibroblast activation, often through ceRNA mechanisms and regulation of miRNA availability. However, a significant limitation is that much of the functional evidence originates from models of DN, ureteral obstruction, or general models of renal fibrosis rather than from models of pure HN. This makes it difficult to attribute the observed effects specifically to hypertension independent of accompanying metabolic abnormalities.

It is worth emphasising that much of the available data on ncRNAs in HN comes from studies of DN or mixed models of kidney injury, which requires careful interpretation and direct comparison of the two diseases.

In both HN and DN, ncRNAs participate in the regulation of key pathophysiological processes, such as inflammation, oxidative stress, endothelial dysfunction, and renal fibrosis. Activation of common molecular axes, including the TGF-β/Smad, PI3K/Akt, and NF-κB pathways, as well as the involvement of similar ncRNA molecules such as miR-21, miR-29, MALAT1, and PVT1, is observed in both diseases. These mechanisms lead to myofibroblast activation, extracellular matrix deposition, and progression of chronic kidney disease.

However, there are significant pathogenetic differences between HN and DN which are reflected in the ncRNA regulatory networks. In DN, the dominant initiating factor is hyperglycaemia and metabolic disturbances, which modulate ncRNA expression through glucose-dependent pathways, insulin resistance, and glycation stress. In contrast, in HN, chronic hemodynamic stress, activation of the RAA system, and endothelial damage associated with impaired NO bioavailability play a key role. Consequently, despite partial overlap between ncRNA profiles, their functional significance and regulatory context may differ.

A particularly interesting and relatively recent group of regulators comprises circRNAs, which, due to their high stability and tissue-specific expression, may represent a link between chronic haemodynamic stress and long-term alterations in gene expression. Studies on models of salt-sensitive hypertension have identified characteristic circRNA signatures in renal tissue, and the identification of the circNr1h4/miR-155-5p/Far1 axis has provided direct evidence of their involvement in the regulation of oxidative stress and redox homeostasis in tubular cells. These findings suggest that circRNAs may function as stable miRNA ‘buffers’ that modulate the intensity of injury signals in hypertension.

Despite promising mechanistic insights, most studies investigating ncRNAs in HN remain preclinical or observational. Direct translation of findings from animal models to human populations is still limited, particularly given the heterogeneity of clinical phenotypes, the duration and control of hypertension, and the presence of comorbidities. An additional challenge is the lack of standardisation in methods used to measure ncRNAs in serum, urine, and EVs, which complicates comparisons across studies and limits assessment of their prognostic value.

In the clinical setting, ncRNAs are emerging as promising biomarkers of early kidney injury and as potential therapeutic targets. However, responses to ncRNA-targeted interventions—as with other molecular therapies—are likely to be highly individualised and dependent on the patient’s genetic, metabolic, and inflammatory background. Furthermore, the long-term safety of ncRNA modulation, particularly given their pleiotropic biological effects, remains insufficiently understood.

Future research should therefore focus on several key priorities:

(1) Precise mechanistic studies to define ncRNA networks that are specific to pure HN.

(2) Well-designed clinical trials with long-term follow-up, including standardised endpoints and validated ncRNA biomarkers.

(3) The development of personalised diagnostic and therapeutic strategies that integrate individual ncRNA profiles with indicators of RAAS activation, oxidative stress, and inflammatory signalling

In summary, ncRNAs—including miRNAs, lncRNAs, and circRNAs—are integral components of the pathogenesis of HN, linking haemodynamic stress with persistent molecular alterations in renal tissue. Improved understanding of these regulatory interactions may not only clarify the mechanisms underlying hypertensive kidney injury but also facilitate more precise diagnostic and therapeutic strategies.

## Figures and Tables

**Table 1:** Selected ncRNA candidates proposed as biomarkers in hypertensive nephropathy and related renal fibrotic contexts.

ncRNA	Results	Sample Type	Model	Clinical Association
miR-21	↑	serum	resistant HTN/HN-related	fibrosis/inflammation
miR-126	↓	serum/urine	hypertensive renal injury	endothelial dysfunction
miR-192	↑	urine	HN/albuminuria-associated	tubular injury/EMT
miR-26a	↓	plasma + urine exosomes	albuminuric hypertensive patients	TGF-β regulation
MALAT1	↑	urinary exosomes/tissue	fibrosis-associated cohorts	fibrosis
hsa_circ_0008925	↑	urinary exosomes	renal fibrosis cohorts	fibrosis burden
hsa_circ_0036649	↑	urinary exosomes	fibrosis cohorts	histologic fibrosis

↑ increased expression, ↓ decreased expression.

## Data Availability

No new data were created or analyzed in this study.

## Abbreviations

The following abbreviations are used in this manuscript:

ASO	Antisense oligonucleotides
AH	Arterial hypertension
ceRNA	Competition endogenous RNA
circRNA	Circular RNA
DN	Diabetic nephropathy
ECM	Extracellular matrix
eGFR	Estimated glomerular filtration rate
EMT	Epithelial–mesenchymal transition
eNOS	Endothelial NO synthase
HN	Hypertensive nephropathy
ITGB1	Integrin β1
LNA	Locked nucleic acid
lncRNA	Long non-coding RNA
MALAT	Metastasis associated lung adenocarcinoma transcript 1
miRNA	MicroRNA
NAT	Natural antisense transcript
ncRNA	Non-coding RNAs
PDTC	Pyrrolidine dithiocarbamate
PTC	Peritubular capillary
RAAS	Renin–Angiotensin–Aldosterone System
RH	Resistant hypertension
ROS	Reactive oxygen species
rRNA	Ribosomal RNAs
TGF-β	Transforming growth factor-β
tRNA	Transfer RNAs
TUG1	Taurine upregulated gene 1
UUO	Unilateral ureteral obstruction
WISP1	Inducible signalling pathway protein 1
α-SMA	α-smooth muscle actin
3′ UTR	3′ untranslated region
